# A Retrospective Study of predicting risk of Metastasis among FDG-avid Bone Lesions in ^18^F-FDG PET/CT

**DOI:** 10.7150/jca.45096

**Published:** 2020-06-21

**Authors:** Guangyu Yao, Yiyi Zhou, Yifeng Gu, Zhiyu Wang, Mengdi Yang, Jing Sun, Quanyong Luo, Hui Zhao

**Affiliations:** 1 Department of Internal Oncology, Shanghai Jiao Tong University Affiliated Sixth People's Hospital, Shanghai, 200030, China.; 2 Department of Radiology, Shanghai Jiao Tong University Affiliated Sixth People's Hospital, Shanghai, 200030, China.; 3 Department of Nuclear Medicine, Shanghai Jiao Tong University Affiliated Sixth People's Hospital, Shanghai, 200030, China.

**Keywords:** Bone metastasis, Biopsy, PET/CT, SUVmax

## Abstract

**Purpose:** We evaluated the imaging and clinical features for discriminating the possibility of metastasis among FDG-avid bone lesions in 18F-FDG PET/CT in patients who have received bone biopsy.

**Methods:** The retrospective study included patients who underwent both ^18^F-FDG PET/CT and bone biopsy for FDG-avid bone lesions. Bone lesions maximum standardized uptake value (SUVmax), CT findings, alongside with common clinical features were analyzed.

**Results:** From the 338 patients enrolled in the final study, all of them were received bone biopsy. Biopsies confirm metastasis in 256 cases (75.74%) and benign tissue in 82 cases (24.26%). Metastasis group had higher bone SUVmax than benign group (median 7.9 vs 4.5, p <0.001). A cutoff bone SUVmax of 5 achieved an AUC of 0.748 in all patients. Lytic CT feature and higher age were more likely frequent in metastasis group. Moreover, in patients without obvious CT abnormality (45, 13.31%), the AUC was 0.743 by a SUVmax cutoff of 5.38, whilst in patients with a solitary bone lesion (74, 21.89%), the AUC was 0.803 by a SUVmax cutoff of 4.3.

**Conclusions:** SUVmax is a promising and valuable metabolic indicator for predicting risk of metastasis among FDG-avid bone lesions in 18F-FDG PET/CT, ancillary clinical and imaging features may increase the probability of a metastatic bone lesion.

## Introduction

Bone metastasis is a common outcome of various types of solid tumors 1, 2, which occurs in 70% cancer patients 3. Skeletal related events (SREs, included spinal cord compression, fracture, bone radiation or surgery, and tumor-related hypercalcemia) are severe and incidental complications after bone metastasis 4. Accurate and prompt diagnosis is therefore crucial for improving patient outcomes.

Traditional screening method for detecting bone metastasis was bone scintigraphy 5. Recently, ^18^F-FDG PET/CT has become a conventional evaluation method 6, with well-defined roles in the diagnosis of solid metastatic tumors 6, as well as suspicious bone lesions 3, 7. Research has shown that ^18^F-FDG PET/CT is superior to bone scintigraphy in detecting bone metastasis 8, 9, which makes PET/CT a promising tool. However, pathological examination is still the gold standard for final diagnosis, which includes CT-guided bone biopsy, open biopsy, and bone surgery 10, 11. In clinical practice, pathological examination is often not carried out 5, particularly in the case of bone metastasis with a definite primary tumor. The reasons are varied: doctors might obtain enough information by only using cancer history and bone imaging features, whilst patients might also refuse pathological examination due to the invasive trauma, additional fees and increased waiting time for pathological result 12.

It is well-known that metabolically active bone lesions on ^18^F-FDG PET/CT could also result from benign bone diseases, such as fracture 13, infection 13, or osteomyelitis 14, 15. When ^18^F-FDG PET/CT scans showed FDG-avid bone lesions, meanwhile bone pathological examination couldn't be performed for the above reasons, confirming the diagnosis and classification of these bone lesions would be meaningful and valuable. As the SUVmax has proven to be a highly repeatable metabolic parameter in oncology 16, it would be of interest to understand whether SUVmax could be a reliable semiquantitative indicator to differentiate a metastatic bone lesion. Consequently, this study aims to determine the best cutoff value of SUVmax to differentiate bone metastasis in ^18^F-FDG PET/CT for the detection of FDG-avid bone lesions in patients who have a final definite diagnosis.

## Materials and Methods

### Patient selection

This retrospective study was approved by the ethics committee of our hospital and formal consent was waived. The Picture Archiving and Communication System (PACS) of our institution was searched for patients with FDG-avid bone lesions who had undergone both ^18^F-FDG PET/CT scans and bone biopsy between December 2010 and March 2018 consecutively. The clinical data of these patients were also collected.

The inclusion criteria were: an ^18^F-FDG PET/CT scan indicating at least one FDG-avid bone lesion (FDG uptake higher than adjacent bones according to the PET/CT reports); a bone biopsy of FDG-avid bone lesion; the ^18^F-FDG PET/CT and pathological confirmation were carried out within one month of each other. The exclusion criteria were: any systemic therapy (such as anti-tumor therapy) between the ^18^F-PET/CT scan and bone biopsy; primary bone tumors confirmed by pathological results; no definite histopathological diagnosis.

### ^18^F-FDG PET/CT

**Figure 1:**
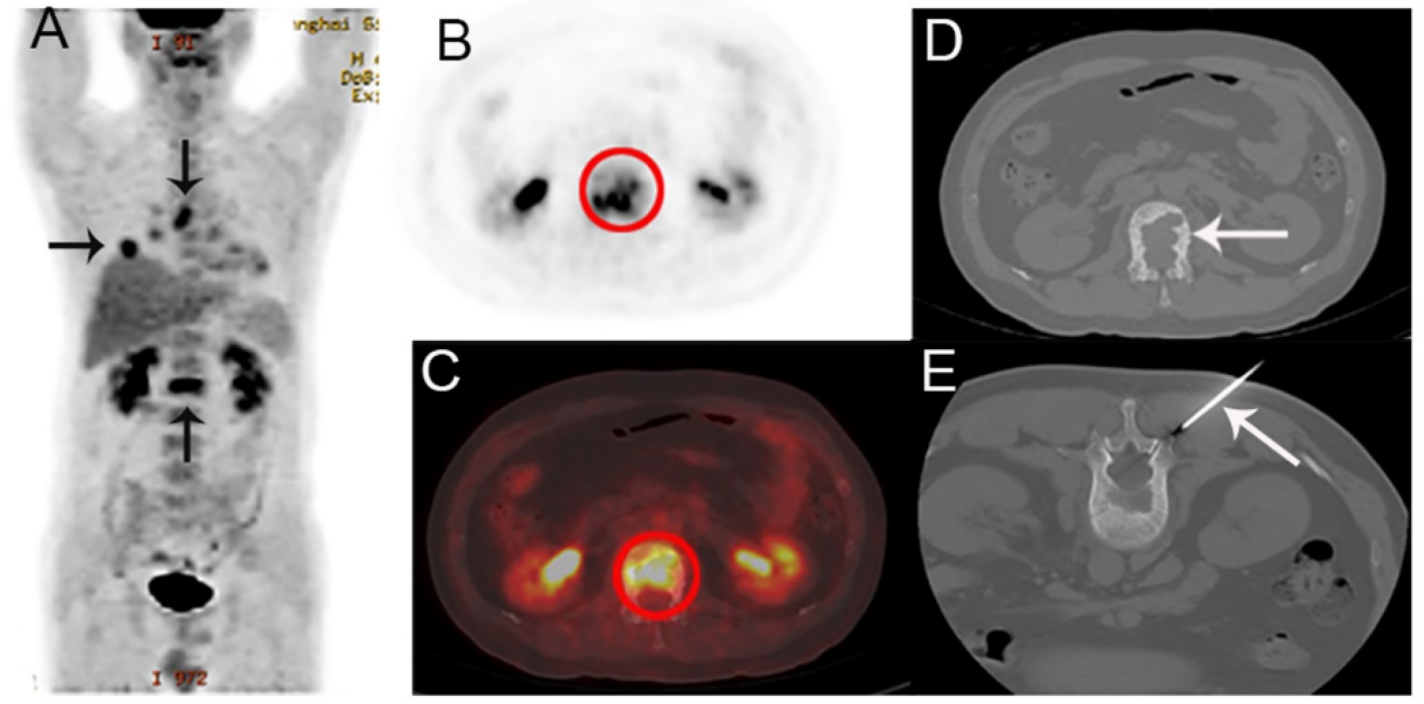
A 44-year-old man presented with back pain, suspected of lung cancer with bone metastasis by PET/CT. A The coronal maximum intensity projection FDG PET image shows multiple FDG-avid lesions in the lung, a mediastinal lymph node and in L1. B,C The axial FDG PET image (B) and fusion images (C) show a FDG-avid lesion (SUVmax 6.7) in L1. D,E The corresponding CT image (D) and the biopsy under CT guidance (E). Histological examination confirmed that the bone lesion was metastatic lung adenocarcinoma. EGFR and ALK were detected as wild and negative, respectively.

**Figure 2:**
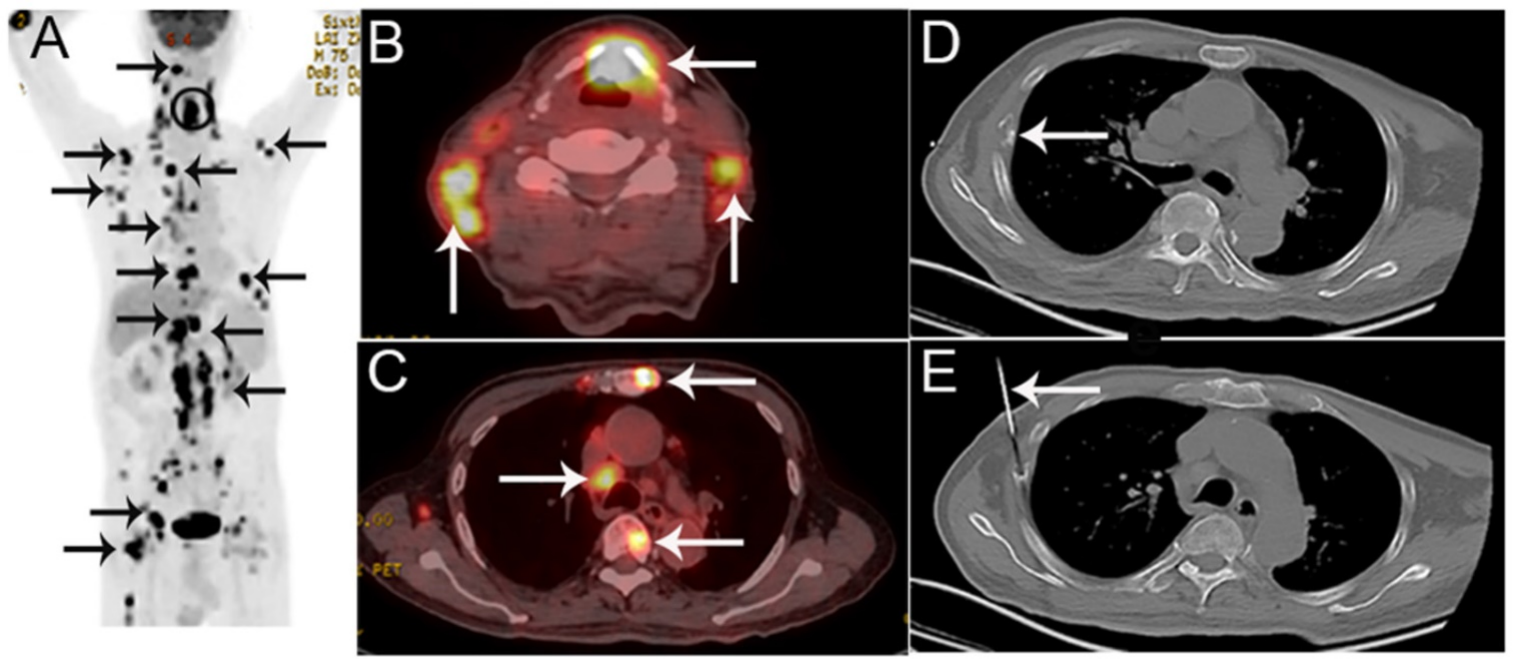
A 75-year-old man presented with hoarseness and hypochondriac pain, suspected of laryngocarcinoma with bone metastasis by PET/CT. A The coronal maximum intensity projection FDG PET image shows multiple FDG-avid lesions in the larynx, lung, extensive lesions in lymph nodes and multiple bone lesions. B,C The fusion images show FDG-avid lesions (SUVmax 13.6) in the larynx, lymph nodes and bone lesions. D,E The corresponding CT image of the 8th rib on the right hand side (D) and the biopsy under CT guidance (E).Histological examination confirmed that the bone lesion was bone tuberculosis, consistent with laryngeal histology.

All patients were required to fast for at least 6h and undergo a peripheral blood sugar test to avoid hyperglycemia. Approximately 1 h after the intravenous injection of ^18^F-FDG [333-518MBq (9-14mCi)], imaging was performed using an integrated PET/CT system (Discovery VCT; GE Medical Systems) from head to lower limbs with the following setting: CT scan, 120 V and 80 mA, 64 slices, with a slice thickness of 3.75mm. PET scans were performed with 2.5 min per bed position. Finally, the CT and PET images were reconstructed iteratively using ordered subset expectation maximization. Attenuation correction was done by unenhanced CT. A senior nuclear medicine doctor then evaluated all of the combined ^18^F-FDG PET/CT scans whilst blinded to the pathological results. The region of interest (ROI) around the bone lesions was drawn on 18F-FDG PET-CT images on each transaxial slice. SUVmax was defined at the peak value on one pixel with the highest counts within the ROI. Representative ^18^F-FDG PET/CT images are shown in Figure 1, and 2.

### Pathological examination

All the included patients were received bone biopsy. The biopsy was performed by an interventional radiologist under CT guidance, with standard procedure made by the department of Radiology. Bone specimens were first decalcified and evaluated by pathologists as our hospital's routine work. Pathological results were considered definite for 327 patients (96.75%) without further exploration. For 11 others with pathological results as bone and cartilage tissue (3.25%), benign diagnoses were confirmed by other imaging modalities (4), bone surgery (2), or follow-up (5).

### Statistical analysis

The characteristics of included patients were compared, using Fisher's exact test for binary data and the Wilcoxon rank-sum test for non-normally distributed continuous data. All tests were two-sided and P values less than 0.05 were considered statistically significant. The above statistical analyses were performed using STATA/IC version 15.1 (StataCorp LLC). The Receiver Operating Characteristic (ROC) curves were drawn by MedCalc version 19.0.4 (MedCalc Software). Then the area under the curve (AUC) was calculated separately, alongside 95% confidence intervals (CI). The cutoff value was determined by the best Youden index on ROC curves analyzed by MedCalc version 19.0.4. All the diagnostic outcomes were on patient-based analysis.

## Results

### Patients

**Figure 3:**
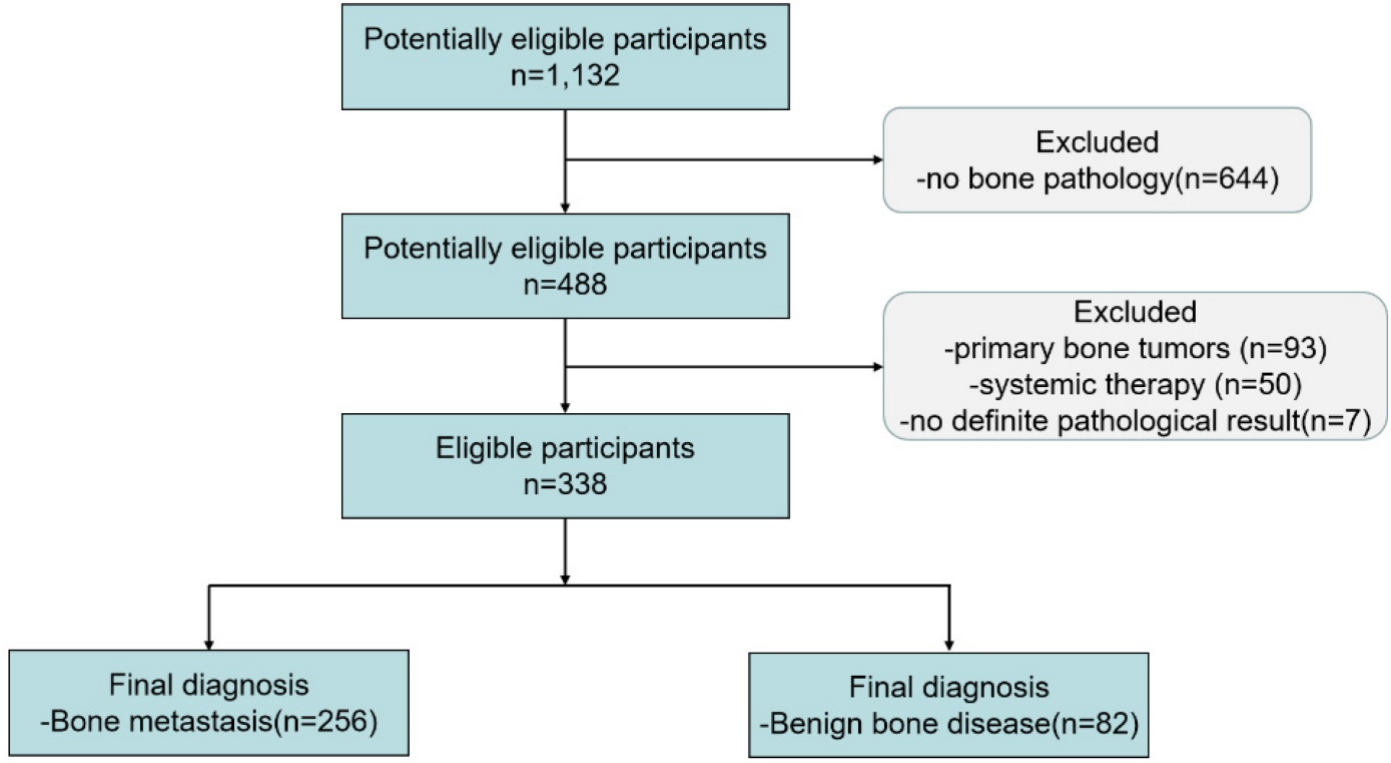
The flowchart of this retrospective study.

Between December 2010 and March 2018, 1,132 patients underwent ^18^F-FDG PET/CT and with FDG-avid bone lesions. 338 patients who met the criteria were included in the final study (see flowchart in Figure 3). From the 338 patients enrolled in the final study, all of them were received bone biopsy. Biopsies confirm metastasis in 256 cases (75.74%) and benign tissue in 82 cases (24.26%).

### Clinical features

The characteristics of included patients were shown in Table 1. The distribution of the final diagnosis was shown in Table 2. In metastasis group, median age was 62 years and 61.33% were male, and the lung cancer metastasis was the most common diagnosis (113/256). Whilst in benign group, median age was 53 years and 45.12% were male, and the bone marrow reaction was the most common diagnosis (17/82).

### Imaging features

**Figure 4:**
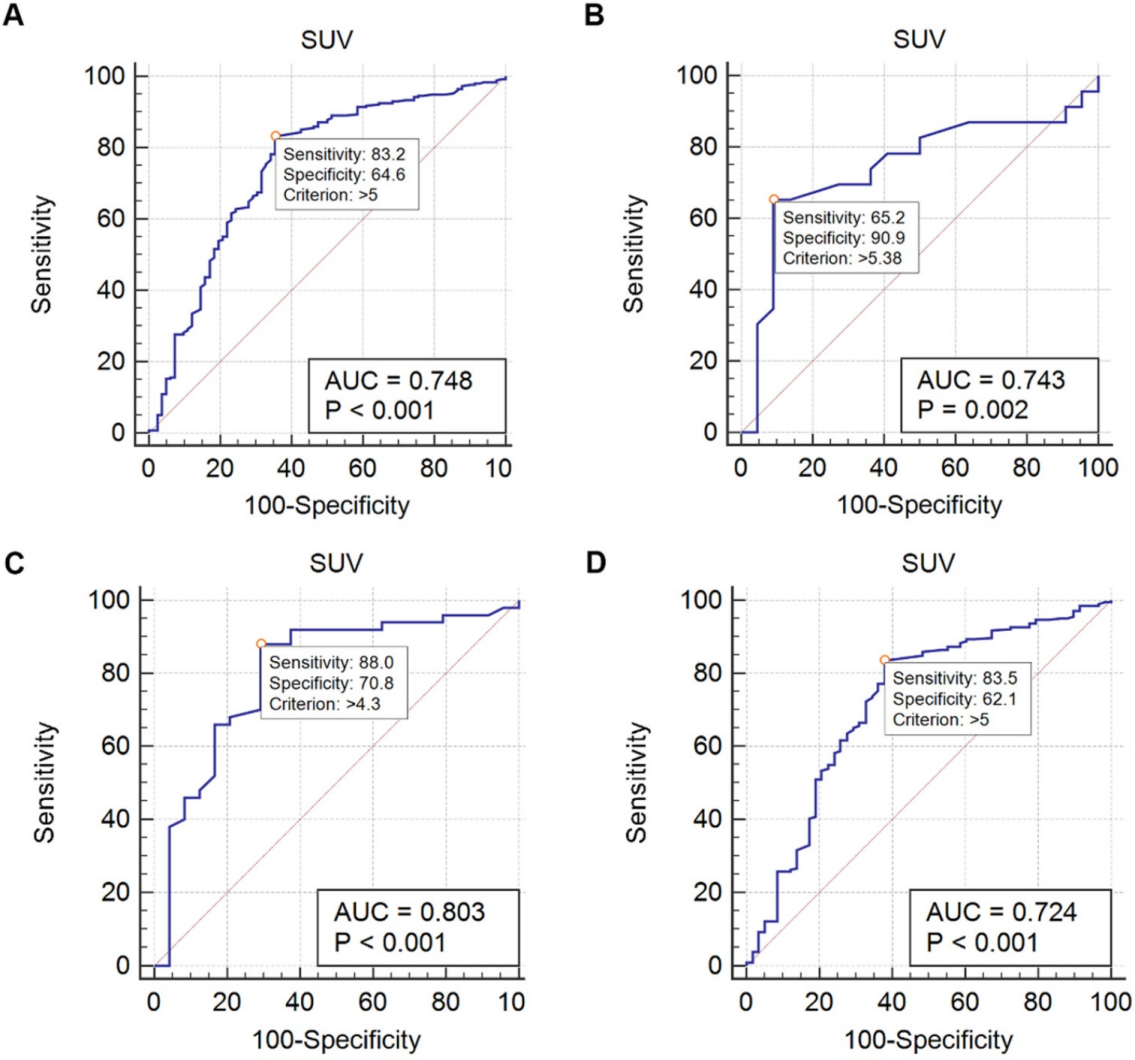
ROC of SUV. A Using a SUVmax threshold of 5, the AUC of predicting bone metastasis is 0.748 (all patients). B Using a SUVmax threshold of 5.38, the AUC of predicting bone metastasis is 0.743 (45 patients with normal CT features). C Using a SUVmax threshold of 4.3, the AUC of predicting bone metastasis is 0.803 (74 patients with a solitary bone lesion). D Using a SUVmax threshold of 5, the AUC of predicting bone metastasis is 0.724 (264 patients with multiple bone lesions).

Metastasis group had higher bone SUVmax than benign group (median 7.9 vs 4.5, p <0.001). ROC curves were drawn to evaluate the differential efficacy of SUVmax. In all 338 patients, the SUVmax 5 showed an AUC of 0.748 to predict bone metastasis. Particularly, in 45 patients without obvious CT abnormality, the AUC was 0.743 by using the SUVmax threshold of 5.38. In 74 patients with only a solitary lesion, the AUC was 0.803 by using the SUVmax threshold of 4.3, whilst in 264 patients with multiple lesions, the AUC was 0.724 by using the SUVmax threshold of 5 (Figure 4, Table 3). For CT findings, lytic CT features were more likely in patients with bone metastasis, whilst CT features without obvious abnormality were more frequent in benign bone disease (p < 0.001, respectively).

## Discussion

Confirming bone metastasis is crucial for the management of successful diagnosis and treatment in cancer patients. In this retrospective study, we examined a group of patients with FDG-avid bone lesions undergoing final pathological confirmations. Our institution is a medical center specializing in various bone diseases. It provides care to patients with suspicious bone malignancies or benign diseases, thus including diverse types of diseases in this study.

Our results showed the substantial differences in characteristics between bone metastasis and benign disease. Male, higher age, higher FDG uptake, lytic lesions were more likely in patients with bone metastasis than benign bone disease (p < 0.001, respectively). Although males seemed more susceptible to bone metastasis, we thought it might be false positive due to the patients' selection. In our study, bone metastasis from breast and prostate cancer were limited, mainly because in clinical practice, doctors may prefer breast or prostate as first pathological site to the metastatic bone because of the convenience and safety. As these two types were undoubtedly gender-related, we should view the difference in our study with reservations. An epidemiologic survey in China exhibited female bone metastasis occupied 53% in contrast to 47% of male 17. Therefore, it's reasonable to decrease the gender difference.

High uptake on PET and changes on CT were typical features of bone metastasis. Taira et al. 18 reported a PPV of 98% for bone metastasis when the findings on PET and CT were both positive. However, there still existed nearly 10% bone metastasis with normal CT features in our study. Recent data has proven PET positive/ CT negative bone lesions were also malignant to a great extent 19, 20. The potential reason for absence of any CT abnormality is the early stage of bone metastasis. When bone marrow metastasis occurred first, structural bone changes hasn't appeared 19, 20. Confirmation with bone biopsy or MRI would be indispensable in this situation.

Previous studies have also investigated this problem 8, 12, 18, 21-23 and to the best of our knowledge, our cohort includes a larger number of patients (338) than any of those studies (between 18 and 202 patients) 8, 12, 18, 21-23. For example, Adams et al. 12 retrospectively reviewed 102 patients who underwent both ^18^F-FDG PET/CT and CT-guided bone biopsy, finding a malignancy PPV of 89.2%. Moreover, Lange et al. 8 used pathological examination as a reference to assess the diagnostic accuracy of imaging methods for skeletal malignancies. In the PET/CT group (58 cases) of this work, the diagnostic characteristics were: sensitivity 92.3%, specificity 63.2%, accuracy 82.7%, PPV 83.7%, and NPV 80%.

Above all, semiquantitative measurements of ^18^F-FDG uptake (SUVmax) were taken, which indicated that SUVmax threshold of 5 could reach an AUC of 0.748 (95% CI 0.698-0.794) to predict bone metastasis. Particularly, in patients without obvious CT abnormality, a cutoff of 5.38 was achieved. Moreover, the cutoff of 4.3 in solitary bone lesion and 5 in multiple lesions were obtained. With these results, we transformed the biometabolic imaging into a semiquantitative analysis, which may make the diagnosis more explicit. An interesting finding was that patients with multiple lesions had a poorer diagnostic performance comparing with solitary bone lesion (AUC 0.724 vs 0.803). One possible explanation was that a substantial proportion of benign bone diseases were systemic diseases which caused lesions limited to a single focus uncommon. As a result, multiple FDG-avid bone lesions were less specific.

Cornelis et al. 24 reported that when percutaneous PET/CT-guided biopsies were carried out on 106 masses, the mean SUVmax was 8.8 and SUVmax > 4 were not significantly more likely to be malignant. However, bone lesions constituted just 31% (33/106) of the massed examined in that study. The majority of studies focusing on bone lesions usually used a threshold SUVmax between 2 and 4 7, which were lower than ours. A reasonable explanation was that the previous studies included many primary bone tumors, whilst our study solely demonstrated on bone metastasis. We believe the heterogeneity in disease characteristics makes it difficult to translate these findings into clinical practice, especially considering the highly variable FDG uptake in primary bone tumors. As a result, we excluded primary bone tumors from the study.

Gomi et al. 25 reported that mean SUVmax of bone metastasis from lung cancer was 7.7. Whist in a study discriminating single-bone FDG lesions in lung cancer 26, a cutoff bone SUVmax of 4.3 was chosen with 81.8% sensitivity, 84.7% specificity, and 83.9% accuracy. The published data above were consistent with ours, which supporting our explanation and results. We believed that our results were persuasive and reliable for two reasons. One was the large sample size; another was using the bone pathological examination as a gold reference, comparing with previous similar studies. These cutoff values could prove extremely helpful when choosing the most suitable site for bone biopsy. Especially when the FDG-avid bone lesion was solitary or normal on CT scanning, SUVmax may be the only clue to make a preliminary diagnosis. After all, it's impractical for a doctor to take biopsies on every suspicious bone lesion; a lesion with the highest potential diagnostic value is the best choice. Two small sample studies (between 20 and 51 patients) 22, 23 had proven PET/CT-guided bone biopsy a promising method in FDG-avid bone lesions.

This study has several limitations. Firstly, lung cancer metastasis comprised over 40% of bone malignancies, which may result in a selection bias. This is likely a reflection of clinical practices, where pathology is carried out at the primary site for other malignancies prone to bone metastases, such as breast, prostate and thyroid cancer. Then a few patients (25/338, 7.39%) underwent bone pathological examination before FDG PET/CT. Ultimately, since this is a retrospective study, we anticipate that prospective trials will be carried out to further evaluate the diagnostic accuracy and clinical utility of SUVmax in FDG-avid bone lesions.

In conclusion, SUVmax is a promising and valuable metabolic indicator for predicting risk of metastasis among FDG-avid bone lesions in ^18^F-FDG PET/CT, ancillary clinical and imaging features may increase the probability of a metastatic bone lesion.

## Figures and Tables

**Table 1:** Basic characteristics of included patients

Characteristic	Bone metastasis	Benign disease	*P* value
Patients	256	82	
**Gender**			
Male, n, (%)	157(61.33)	37(45.12)	0.011^a^
Female, n, (%)	99(38.67)	45(54.88)	
**Age (yeas)**			
Mean	60.1	52.5	
Median	62	53	<0.001^b^
**Bone lesion**			
Solitary, n, (%)	50(19.53)	24(29.27)	0.067 ^a^
Multiple, n, (%)	206(80.47)	58(70.73)	
Mean SUVmax	8.9	6.0	
Median SUVmax	7.9	4.5	<0.001^b^
**CT features**			
Lytic, n, (%)	179(69.92)	13(15.83)	<0.001^a^
Normal, n, (%)	23(8.98)	22(26.83)	<0.001^a^
Extraskeletal lesion, n, (%)	181(70.70)	57(69.51)	0.890 ^a^
**Bone pathology site, n, (%)**	256(100)	82(100)	
Vertebra	77(30.08)	18(21.95)	0.162 ^a^
Pelvis	86(33.59)	33(40.24)	0.290 ^a^
Extremity	75(29.30)	26(31.71)	0.680 ^a^
Others	18(7.03)	5(6.10)	1.000 ^a^
PET/CT before bone pathology, n, (%)	234(91.41)	79(96.34)	0.223 ^a^
**Interval between PET/CT and bone biopsy**			
Median (days)	5	4.5	0.159 ^b^
Range (days)	0-31	0-31	

^a^ Fisher's exact test; ^b^ Wilcoxon rank-sum (Mann-Whitney) test.

**Table 2:** Distribution of the final diagnoses (Top 6 each)

Final diagnoses	No
Bone metastasis	
Lung cancer metastasis	113
Digestive tumor metastasis	48
Hematological malignancy metastasis	27
Breast cancer metastasis	18
Thyroid cancer metastasis	11
Kidney cancer metastasis	11
Benign bone disease	
Bone marrow hyperplasia or normal bone marrow	17
Inflammation/Infection of unknown origin	16
Bone and cartilage tissue	11
Fracture	6
Osteomyelitis	6
Tuberculosis	5

**Table 3:** Diagnostic characteristics of SUVmax

Diagnostic outcomes	All patients (338)	Normal CT features (45)	Solitary (74)^a^	Multiple (264)^b^
Cutoff value	5	5.38	4.3	5
Sensitivity	83.2%	65.2%	88.0%	83.5%
Specificity	64.6%	90.9%	70.8%	62.1%
AUC	0.748	0.743	0.803	0.724
95%CI	0.698-0.794	0.591-0.862	0.694-0.886	0.666-0.777

^a^ Solitary means a solitary bone lesion on PET/CT; ^b^ Multiple means multiple bone lesions on PET/CT.

## References

[1] Li S, Peng Y, Weinhandl ED, Blaes AH, Cetin K, Chia VM (2012). Estimated number of prevalent cases of metastatic bone disease in the US adult population. Clin Epidemiol.

[2] Coleman RE, Lipton A, Roodman GD, Guise TA, Boyce BF, Brufsky AM (2010). Metastasis and bone loss: advancing treatment and prevention. Cancer Treat Rev.

[3] Schmidkonz C, Ellmann S, Ritt P, Roemer FW, Guermazi A, Uder M (2019). Hybrid Imaging (PET-Computed Tomography/PET-MR Imaging) of Bone Metastases. PET Clinics.

[4] von Moos R, Costa L, Gonzalez-Suarez E, Terpos E, Niepel D, Body JJ (2019). Management of bone health in solid tumours: From bisphosphonates to a monoclonal antibody. Cancer Treat Rev.

[5] Shibata H, Kato S, Sekine I, Abe K, Araki N, Iguchi H (2016). Diagnosis and treatment of bone metastasis: comprehensive guideline of the Japanese Society of Medical Oncology, Japanese Orthopedic Association, Japanese Urological Association, and Japanese Society for Radiation Oncology. ESMO Open.

[6] Podoloff DA, Ball DW, Ben-Josef E, Benson AB 3rd, Cohen SJ, Coleman RE (2009). NCCN task force: clinical utility of PET in a variety of tumor types. J Natl Compr Canc Netw.

[7] Parghane RV, Basu S (2017). Dual-time point (18)F-FDG-PET and PET/CT for Differentiating Benign From Malignant Musculoskeletal Lesions: Opportunities and Limitations. Semin Nucl Med.

[8] Lange MB, Nielsen ML, Andersen JD, Lilholt HJ, Vyberg M, Petersen LJ (2016). Diagnostic accuracy of imaging methods for the diagnosis of skeletal malignancies: A retrospective analysis against a pathology-proven reference. Eur J Radiol.

[9] Liu NB, Zhu L, Li MH, Sun XR, Hu M, Huo ZW (2013). Diagnostic value of 18F-FDG PET/CT in comparison to bone scintigraphy, CT and 18F-FDG PET for the detection of bone metastasis. Asian Pac J Cancer Prev.

[10] Lukaszewski B, Nazar J, Goch M, Lukaszewska M, Stepinski A, Jurczyk MU (2017). Diagnostic methods for detection of bone metastases. Contemp Oncol (Pozn).

[11] Huang AJ, Kattapuram SV (2011). Musculoskeletal neoplasms: biopsy and intervention. Radiol Clin North Am.

[12] Adams HJ, de Klerk JM, Heggelman BG, Dubois SV, Kwee TC (2016). Malignancy rate of biopsied suspicious bone lesions identified on FDG PET/CT. Eur J Nucl Med Mol Imaging.

[13] Lemans JVC, Hobbelink MGG, FFA IJ, Plate JDJ, van den Kieboom J, Bosch P (2019). The diagnostic accuracy of (18)F-FDG PET/CT in diagnosing fracture-related infections. Eur J Nucl Med Mol Imaging.

[14] Govaert GA, FF IJ, McNally M, McNally E, Reininga IH, Glaudemans AW (2017). Accuracy of diagnostic imaging modalities for peripheral post-traumatic osteomyelitis - a systematic review of the recent literature. Eur J Nucl Med Mol Imaging.

[15] Kouijzer IJE, Scheper H, de Rooy JWJ, Bloem JL, Janssen MJR, van den Hoven L (2018). The diagnostic value of (18)F-FDG-PET/CT and MRI in suspected vertebral osteomyelitis - a prospective study. Eur J Nucl Med Mol Imaging.

[16] Lodge MA (2017). Repeatability of SUV in Oncologic (18)F-FDG PET. J Nucl Med.

[17] Yang Y, Ma Y, Sheng J, Huang Y, Zhao Y, Fang W (2016). A multicenter, retrospective epidemiologic survey of the clinical features and management of bone metastatic disease in China. Chin J Cancer.

[18] Taira AV, Herfkens RJ, Gambhir SS, Quon A (2007). Detection of bone metastases: assessment of integrated FDG PET/CT imaging. Radiology.

[19] Al-Muqbel KM (2017). Bone Marrow Metastasis Is an Early Stage of Bone Metastasis in Breast Cancer Detected Clinically by F18-FDG-PET/CT Imaging. BioMed Research International.

[20] Garg G, DaSilva R, Kim M, Love C, Abraham T (2017). Relevance of focal osseous uptake on FDG PET with or without CT changes in oncology patients. Clin Imaging.

[21] Schulte M, Brecht-Krauss D, Heymer B, Guhlmann A, Hartwig E, Sarkar MR (2000). Grading of tumors and tumorlike lesions of bone: evaluation by FDG PET. J Nucl Med.

[22] Klaeser B, Wiskirchen J, Wartenberg J, Weitzel T, Schmid RA, Mueller MD (2010). PET/CT-guided biopsies of metabolically active bone lesions: applications and clinical impact. Eur J Nucl Med Mol Imaging.

[23] Guo W, Hao B, Chen HJ, Zhao L, Luo ZM, Wu H (2017). PET/CT-guided percutaneous biopsy of FDG-avid metastatic bone lesions in patients with advanced lung cancer: a safe and effective technique. Eur J Nucl Med Mol Imaging.

[24] Cornelis F, Silk M, Schoder H, Takaki H, Durack JC, Erinjeri JP (2014). Performance of intra-procedural 18-fluorodeoxyglucose PET/CT-guided biopsies for lesions suspected of malignancy but poorly visualized with other modalities. Eur J Nucl Med Mol Imaging.

[25] Gomi D, Fukushima T, Kobayashi T, Sekiguchi N, Koizumi T, Oguchi K (2019). Fluorine-18-fluorodeoxyglucose-positron emission tomography evaluation in metastatic bone lesions in lung cancer: Possible prediction of pain and skeletal-related events. Thorac Cancer.

[26] Lim CH, Ahn TR, Moon SH, Cho YS, Choi JY, Kim BT (2019). PET/CT features discriminate risk of metastasis among single-bone FDG lesions detected in newly diagnosed non-small-cell lung cancer patients. Eur Radiol.

